# Enhancing Disaster Resilience by Reducing Stress-Associated Health Impacts

**DOI:** 10.3389/fpubh.2018.00373

**Published:** 2018-12-21

**Authors:** Paul A. Sandifer, Ann Hayward Walker

**Affiliations:** ^1^Center for Coastal Environmental and Human Health, School of Sciences and Mathematics, College of Charleston, Charleston, SC, United States; ^2^Center for Oceans and Human Health, University of South Carolina, Columbia, SC, United States; ^3^SEA Consulting Group, Cape Charles, VA, United States

**Keywords:** disasters, resilience, stress, health, well-being, hurricanes, oil spills

## Abstract

Disasters are a recurring fact of life, and major incidents can have both immediate and long-lasting negative effects on the health and well-being of people, communities, and economies. A primary goal of many disaster preparedness, response, and recovery plans is to reduce the likelihood and severity of disaster impacts through increased resilience of individuals and communities. Unfortunately, most plans do not address directly major drivers of long-term disaster impacts on humans—that is, acute, chronic, and cumulative stress—and therefore do less to enhance resilience than they could. Stress has been shown to lead to or exacerbate ailments ranging from mental illness, domestic violence, substance abuse, post-traumatic stress disorders, and suicide to cardiovascular disease, respiratory problems, and other infirmities. Individuals, groups, communities, organizations, and social ties are all vulnerable to stress. Based on a targeted review of what we considered to be key literature about disasters, resilience, and disaster-associated stress effects, we recommend eight actions to improve resiliency through inclusion of stress alleviation in disaster planning: (1) Improve existing disaster behavioral and physical health programs to better address, leverage, and coordinate resources for stress reduction, relief, and treatment in disaster planning and response. (2) Emphasize pre- and post-disaster collection of relevant biomarker and other health-related data to provide a baseline of health status against which disaster impacts could be assessed, and continued monitoring of these indicators to evaluate recovery. (3) Enhance capacity of science and public health early-responders. (4) Use natural infrastructure to minimize disaster damage. (5) Expand the geography of disaster response and relief to better incorporate the displacement of affected people. (6) Utilize nature-based treatment to alleviate pre- and post-disaster stress effects on health. (7) Review disaster laws, policies, and regulations to identify opportunities to strengthen public health preparedness and responses including for stress-related impacts, better engage affected communities, and enhance provision of health services. (8) With community participation, develop and institute equitable processes pre-disaster for dealing with damage assessments, litigation, payments, and housing.

## Introduction

*Homo sapiens* and their ancestors have been dealing with disasters since thinking beings first appeared on Planet Earth. In the beginning, these were primarily disasters of the natural world, but as time progressed more and more have been human-influenced or human-caused (1). Because disasters have been such an important and continuing part of human existence, highlighted by periodic devastating consequences, individuals, communities, and societies have poured more and more effort into learning how to cope with them, including preparation, response, and recovery activities. In North America, these became more organized and systematized during the Cold War years as a result of threats of nuclear war (2). Preparedness efforts have become much more sophisticated over time as society has attempted to incorporate some of the lessons learned from each disaster. However, disasters and their destructive effects continue to have huge and perhaps increasing impacts on human populations today. In this paper, we briefly review what disasters are, effects of recent disasters on human health and well-being, the concept of resiliency, ways resiliency applies to improving human capabilities to deal with disasters, and the pernicious health effects of disaster-caused stress, in order to elucidate how a primary focus on reducing negative stress-associated health outcomes could improve individual and community resilience to disasters. We use the World Health Organization's definition of health: “a state of complete physical, mental and social well-being and not merely the absence of disease or infirmity” (3). The Millennium Ecosystem Assessment (4) further elaborated the concept of human well-being to include not only health, but also supporting ecological and social environments, security, income, and opportunity for education. For this paper, we conducted a targeted literature review, with papers selected based on a knowledgeable assessment of the literature and explicit relevance to the issues addressed. This targeted review also explored existing work of practitioners with consideration that future improvements should leverage the collective insights gained from research findings, practice-based knowledge, and experience with communities and disasters. The resulting recommendations provided here not only provide a roadmap for enhancing resiliency, but also should provide a platform to launch future studies to address stress-associated vulnerabilities in resilience planning.

## Defining the Disaster Landscape

In chapter 1 of the new *Handbook of Disaster Research*, Perry (5) discusses the evolution of disaster definitions from the point of view of theory-based social science research. He concludes that “most researchers currently view social disruption as the key defining feature or essential dimension” of disasters. The United Nations International Strategy for Disaster Reduction (UNISDR) defines disaster as: “A serious disruption of the functioning of a community or a society at any scale due to hazardous events interacting with conditions of exposure, vulnerability and capacity, leading to one or more of the following: human, material, economic and environmental losses and impacts” (6). Gill and Ritchie (7) describe a useful typology for disasters, grouping them into two general categories, natural and technological (Table 1), with further elaboration of connected events as follows:

(1) Natural disasters are acts of nature (or God); they occur naturally, are predictable to an extent, but are not preventable although their impacts can be managed or mitigated to a degree. No one causes these disasters; they occur *naturally* and are arguably the most common. Examples include hurricanes, droughts, tornadoes, high winds, coastal, and inland flooding, landslides, earthquakes, temperature extremes (high and low), and naturally caused and climate-related wildfires (8), plus volcanic eruptions and tsunamis.
(2) Technological disasters are acts of *humans* that result from malfunction of human-designed technology, human error, regulatory failure, and/or management shortcomings. These have a detectable cause and an identifiable party who can (theoretically at least) be held responsible for some of the damages caused by the disaster. Technological disasters are characterized by recreancy (9), a term that encompasses institutional failures and societal loss of trust in individuals and organizations which should have managed risks to prevent these disasters.
(3) Natech disasters are those where a natural disaster such as a hurricane or flood leads to a technological disaster such as an oil or other chemical spill, a dam failure, or a nuclear reactor meltdown. To the degree to which humans are a cause of ongoing climate change, major negative effects of climate change can be considered Natech disasters.
(4) Techna disasters are those where a failure or unanticipated consequence of human-designed technology leads to or exacerbates a natural hazard, such as increased earthquake activity related to fracking processes or where failure to establish or follow appropriate construction practices increases the property destruction and injuries to people from earthquakes.

**Table 1:** Comparison of natural and technological disaster characteristics [from Gill and Ritchie (7), used with permission].

**Natural disasters**	**Technological disasters**
**ETIOLOGY/ORIGINS**	
■ Rooted in nature—meteorological, geological, hydrological, biological ■ Often predictable—geographic location, seasonality, frequency ■ Not preventable ■ Associated with perceived *lack* of control	■ Caused by humans—identifiable parties to hold accountable ■ Result of technological malfunctions, human error, or “recreancy” ■ Not predictable but perceived to be preventable ■ Associated with perceived *loss* of control
**PHYSICAL DAMAGES**	
■ Casualties—deaths & injuries ■ Visible damage to the built environment (e.g., lifelines, buildings, roads, bridges) ■ Assess damages in monetary and other quantifiable terms ■ Consensus regarding damage	■ Environmental contamination and toxic exposure are relatively invisible ■ Uncertainty regarding extent & nature of the damage—“ambiguity of harm” ■ Contested interpretations of damages
**DISASTER PHASES**	
■ Preparedness (planning and warning) ■ Response (pre-impact and post-impact) ■ Recovery (restoration and reconstruction) ■ Mitigation (hazard perceptions and adjustments)	■ Difficult to pinpoint a beginning and an end—lack of finality/closure ■ Those affected often enter into a corrosive warning, threat, impact, & blame cycle with no clear path to recovery ■ “Secondary traumas” emerge and may become chronic
**POST-DISASTER PROCESSES**	
■ Agency & organization support & responses ■ Stafford Act ■ Insurance claims, low interest loans	■ Compensation for “legitimate” claims ■ Litigation (typically adversarial & protracted) against the primary responsible party ■ Prompts reexamination of government policies and new legislation
**VULNERABILITY**	
■ Sociodemographic—age, gender, race/ethnicity, class, special needs populations ■ Geographic or place-based—exposure to natural hazards ■ Exposure—disaster experience, damages, & losses ■ Limited access to social & political capital	■ Individuals potentially vulnerable irrespective of traditional sociodemographic characteristics ■ Geographic or place-based—proximity to technological hazards; environmental justice issues ■ Exposure to toxins—amount, duration, & type ■ Sociocultural & psychosocial relationships with the natural environment
**COMMUNITY REACTIONS**	
■ “Therapeutic” or “altruistic” community emerges; communities experience “post-disaster utopia” and “amplified rebound” ■ Collective definition of the situation—“community of sufferers” ■ “Lifestyle change” ■ Initial local response	■ “Collective trauma” and emergence of a “corrosive community” ■ No collective definition of the situation—individuals forced to create their own ■ Social capital loss spirals ■ “Lifestyle change” and “lifescape change” ■ Grassroots responses
**INDIVIDUAL REACTIONS**	
■ Short-term psychosocial stress and social disruption ■ Immediate, acute health impacts & injuries	■ Short-term and chronic psychosocial stress & social disruption ■ Prolonged uncertainty ■ Reluctant resignation ■ Long-term negative health outcomes

All of these unintentional types of disasters include social disruption as a major element and consequence. Intentional acts of arson (e.g., wildfires), mass shootings, and terrorism can become disasters with dramatic impacts on the communities which they impact.

## Recent History of Disasters

The U.S. has experienced 230 weather- or climate-related (“natural”) disasters that each exceeds $1 Billion in damages since 1980, with a total economic cost of $1.5 Trillion (10). These include hurricanes and other severe storms, tornados, droughts, freezes, wildfires, etc. Examples from recent years stand witness to the magnitude of the problem. In 2005, the U.S. suffered ~$160 B in disaster damage, of which some $85 B as well as >1,800 deaths were the result of Hurricane Katrina alone. In 2010, the catastrophe of the explosion aboard the Deepwater Horizon oil rig led to the single largest technologically-based environmental disaster in US history, known as the Deepwater Horizon (DWH) oil spill with the responsible parties (RPs), i.e., the U.S. term for polluters, paying some $62 B to address damages to the environment and economy (11). In 2012, the U.S. sustained approximately $110 B in damages and 377 deaths from 11 major weather disasters including Hurricane Sandy. And 2017 brought the highest costs to date for weather- and climate-associated disasters, some $306 B, from sixteen > $1 B impact events/event groups (10). Just three incidents—Hurricanes Harvey, Irma, and Maria—accounted for 86.6% of the economic losses and the large majority of deaths. In Texas, Hurricane Harvey interrupted power supply to 300,000 people, affected > 119,000 homes, damaged some 500,000 automobiles, caused major industrial and infrastructure damage, and resulted in 78 direct and indirect deaths (12). Irma was the strongest Atlantic hurricane ever recorded (13). It slammed into Barbuda with terrible intensity and then hit Puerto Rico and savaged the Florida Keys and other areas of South Florida, killing over 100 people and causing widespread damage. Irma was followed closely by Maria, which devastated the US Virgin Islands and all of Puerto Rico, leaving nearly the entire population of Puerto Rico, 3.7 million people, without power, many for a very long period of time following landfall. The number of deaths in Puerto Rico directly and indirectly attributable to Maria is still uncertain, but likely to be between 1,400 (14) and 5,000 (15), where the latest estimate of 2,975 (16) falls. All of these are many times the original official estimate of 64 deaths caused directly by the storm. Together, these three storms plus the 2017 California wildfires resulted in more households filing for federal assistance (4.8 million as of May 2018) than the combined total for the previous decade (17). Not surprisingly, the Federal Emergency Management Agency (FEMA) labeled the 2017 disaster year in the U.S. as “unprecedented in scale, scope, and impact.”

**Figure 1:**
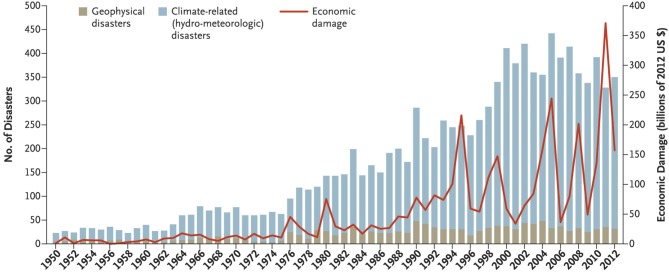
Numbers and types of natural disasters, 1950–2012, not including biological disasters [From Learning and Guha-Sapir (18). Massachusetts Medical Society. Reprinted with permission from Massachusetts Medical Society].

On a global basis, the long-term trend shows the number of climate- and weather-related disasters has more than doubled over the past four decades, and these accounted for a majority of disaster deaths in most years (Figure 1) (18, 19). While improved tracking and communication may account for some of the increase, Learning and Guha-Sapir (18) noted that “the growth is mainly in climate-related events, accounting for nearly 80% of the increase, whereas trends in geophysical events have remained stable.”

**Figure 2:**
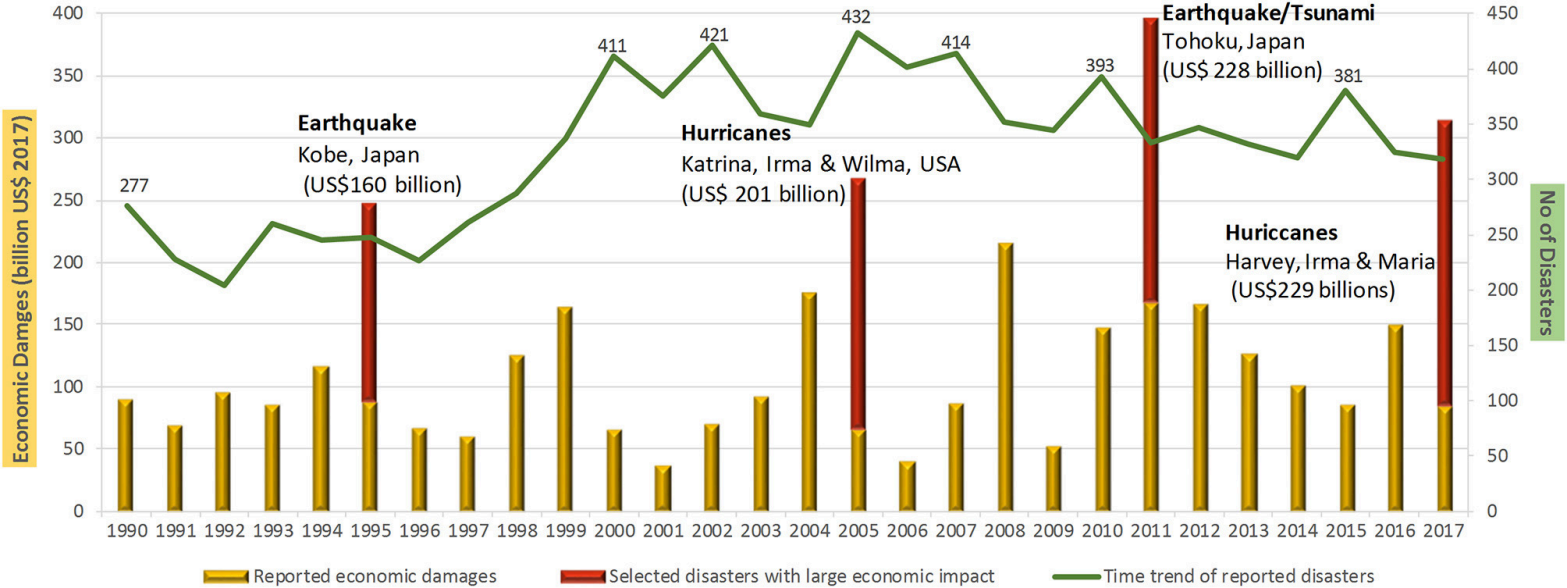
Annual occurrence and economic damages from natural disasters 1990–2017 [reprinted with permission from CRED (20)].

Some good news is that over the past few years the number of storms causing deaths may be decreasing; however, the “deadliness” of individual storms may be rising. Overall, in 2016 only 342 natural disasters were recorded compared to the 20-year average of 376 and considerably down from the peak of 395 in 2015 (6). Interestingly, 2017 saw another decline to 318 (20) (Figure 2). These statistics may signal a return to lower occurrence levels; however, data from just 2 years are not enough to suggest a change in the long- term trend that still shows much higher incidence than in previous decades (Figures 1, 2). There also appear to be reductions in total mortalities associated with natural disasters, perhaps as a matter of better knowledge and preparation. Nevertheless, the economic impacts continue to rise as does the severity of individual events. Overall, CRED (20) reported that “weather related disasters were responsible for the majority of both human and economic losses in 2017.” Technological disasters, which were not included in the Learning and Guha-Sapir (18) or CRED (20) summaries, may be increasing, at least in scale and impact, with most of the worst (such as the Bhopal, Chernobyl, DWH oil spill and Fukushima accidents) having occurred over the past 50 years or so (21–24).

## Disasters and Resilience

As occurrences, intensity and impacts of extreme natural and technological disasters continue to worsen, the need to increase disaster resilience among individuals, groups (e.g., families, work groups, civic organizations, churches), and especially communities, has become a common focus of scholarly papers about disasters (25, 26). While the concept of resiliency related to disasters has been around for a long time, definitions and interpretations of exactly what disaster resilience means are numerous, and there is no consensus as yet among disaster researchers (8, 25, 26). From a natural science point of view, the concept of resilience harks back at least to the work of Holling (27) on ecological resilience. In the ecological context, Darling and Cote (28) noted that “The term ‘resilience’ captures two dynamic processes: the ability of ecosystems to resist and absorb disturbance, and their ability to recover.” Similarly, Ostadtaghizadeh et al. (29) posed a definition of community resilience that is now used by the UNISDR as “the ability of a system, community, or society exposed to hazards to resist, absorb, accommodate to and recover from the effects of a hazard in a timely and efficient manner through the preservation and restoration of its basic structures and functions.”

Resilience is a logical concept which speaks to something everyone wants—the ability to bounce back from a serious, disruptive event, and resume a productive life going forward. Despite some serious criticisms of the value of the resilience concept [discussed in Kendra et al. (26)], it is widely accepted as useful in planning for response to disasters. Over the past two decades, resilience has become enshrined in US national science and policy frameworks. Several high-level examples include disaster-focused National Science Foundation research programs (https://www.nsf.gov/naturaldisasters/), President George W. Bush's 2007 Homeland Security Presidential Directive 21 on Public Health and Medical Preparedness (30), the 2010 Quadrennial Homeland Security Report (31), President Obama's 2011 Presidential Directive on National Preparedness (32), the 2011 Centers for Disease Control (CDC) Public Health Preparedness Capabilities National Standards for State and Local Planning (33), and FEMA's (34) “whole community approach to emergency management” report. Kendra et al. (26) point out that: (1) most disaster-related resilience work focuses on communities; (2) at a foundation level, resilience is part of “[society's] ongoing search for survival;” and (3) while social capital “is at the forefront of thinking about community resilience,” economic, human, institutional, political, and community capital, improvisation, natural resources, and physical resources are also key elements. Surprisingly, human health and well-being are not mentioned by Kendra et al. (26) as a key element *per se*, but some aspects of health or health care are included in the more detailed descriptions of community capital, human capital, social capital, and physical infrastructure. Kendra et al. (26) also reviewed several different models for assessing or measuring resilience, primarily at the community level. Two notable examples are the Resilience Activation Framework (35) developed following the DWH oil spill and the Composite of Pre-Event Well-being (COPEWELL) model (36). The COPEWELL model includes health and health care along with well-being among its 10 domains of pre-event community functions.

Walker (37) described several different forms of resilience related to oil spill response activities (Table 2), including formal resilience (also referred to as planned institutional resilience and formal contingency plans), meaning specific “predetermined planning and capabilities” encouraged or implemented through government or private business entities (38, 39), and inherent and adaptive resilience that results from locally-based capacities to cope with disruption (38). In a systematic review of the term “community resilience,” Patel et al. (25) distilled nine “core elements” of community resilience from 80 relevant papers: local knowledge, community networks and relationships, communication, health (emphasis added), governance and leadership, economic investment, resources, preparedness, and mental outlook. These authors noted that the community resilience concept has a strongly positive connotation, but focusing on individual components, like health, as suggested here, may be more effective for implementation, particularly in supporting adaptive resilience, that is the ability to adapt to as well as recover from disaster situations.

**Table 2:** Examples of oil spill activities and elements of resilience [from Walker (37), used with permission, adapted from Colten et al. (38)].

**Form of Resilience**	**Risk Anticipation (Preparedness)**	**Reduce Vulnerability(Pre-spill and Emergency Phase)**	**Response**	**Recovery**
**Formal Resilience:** Government	• Contingency plans • Response organization, e.g., national contingency plan structure • Spill control organizations	• Training • Implement pollution contingency plans • Monitoring for public health and worker safety • Close fisheries • Monitor seafood quality	• Oversight of response through incident management teams (IMTs) • Biological analysis • Post-spill legislation • Alternate employment programs	• Post-spill improvements to regulations or new legislation • Compensation program; e.g., claims and natural resource damage assessment • Implement incident learnings
**Formal Resilience:** Potential Responsible Parties (Polluters)	• Conduct operational risk assessments • Arrangements with spill control organizations • Develop spill response/accident contingency plans, e.g., with blowout prevention	• Regulatory compliance • Develop/implement response/ contingency plans • Training	• Source control, e.g., cap well, pollutant monitoring, Skimming, burning, boom, dispersants, beach clean-up	• Implement incident learnings • Marketing to promote seafood and tourism in an affected area • Settlements
**Inherent and Adaptive Resilience:** Community/Family	• Participation in development of community-level spill contingency/ emergency plans, e.g., natural and socio-economic resource protection strategies	• Joint training with oil spill planners and responders • Community liaison representatives with the IMTs • IMT safety and health connections with community health workers • Advisory participation in pollution-related emergency fishery management, fishery closures and fishery openings	• Assist with monitoring of extent of contamination • Volunteers • Family aid • Strategies for alternative fishing locations/approaches • Personal economic diversification • Relocate	• Participate in restoration process, e.g., input to setting priorities for recovery actions • Receiving compensation from law suits • Receiving unemployment compensation • Conduct peer-listening

## Disaster Health Effects Associated with Stress

**Figure 3:**
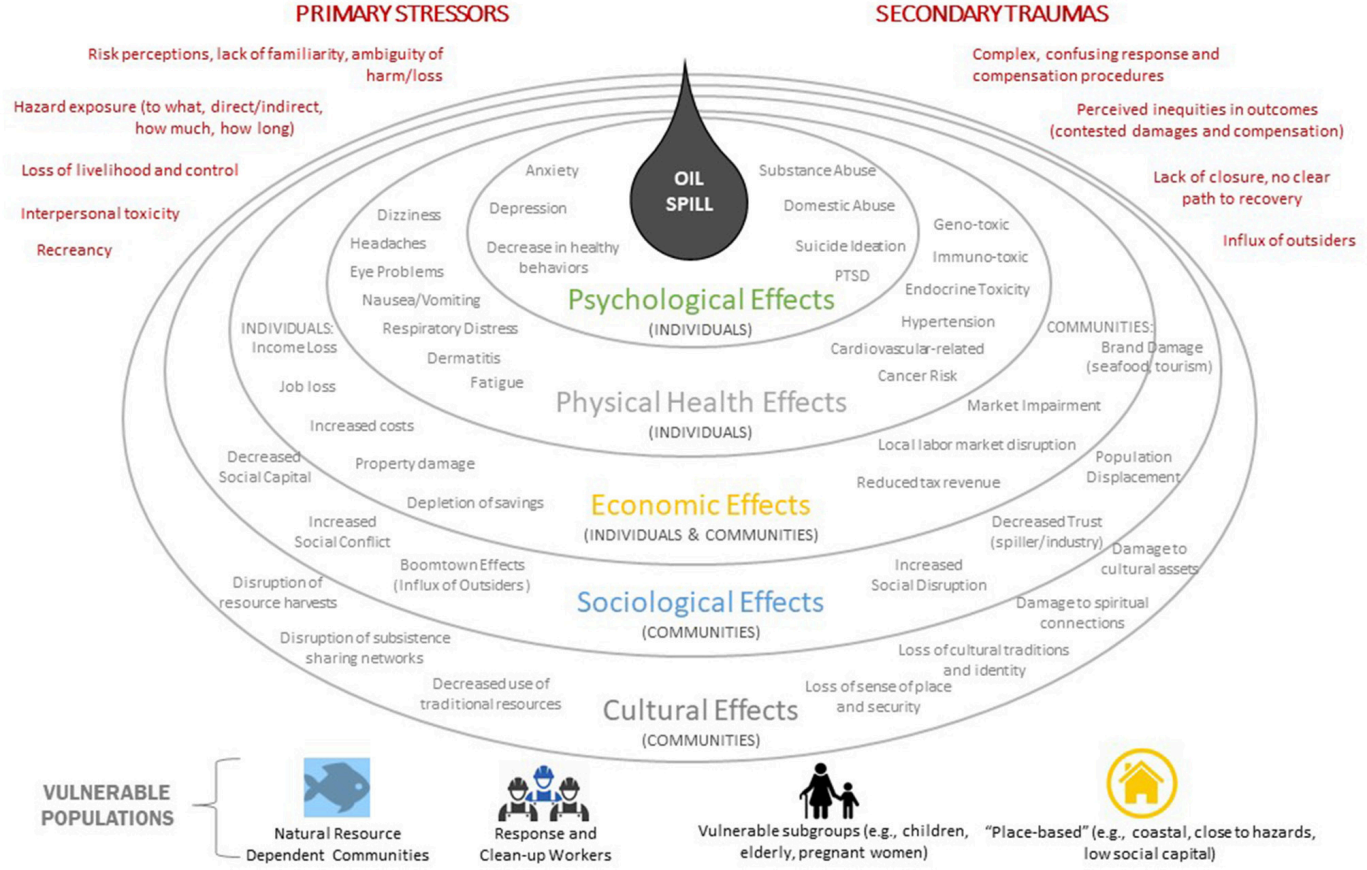
Array of human effects which have been reported from past oil spills. Whether effects could occur, as well as type and scale, depends upon actual spill conditions and location. Effects may be modified by re-existing conditions, vulnerabilities, previous disaster experience, and other factors. Developed by Nicholls et al. (40). Modeled after Bayer et al. (41).

Oil spills and other kinds of disasters can result in a wide range of interrelated effects on humans and human communities (Figure 3). The most pernicious and persistent human health and well-being impacts of disasters may be those related to stress, both mental and physical (11, 42). Stress has been shown to lead to or exacerbate a wide range of both mental illness and physical disorders, including such things as anxiety, depression, post-traumatic stress disorder, respiratory problems, cardiovascular disease, and many others (Table 3). Individuals, groups, communities, and even organizations and social ties are all vulnerable to stress-related or stress- caused negative effects. Chronic stress and its negative effects can be related to disaster-associated injury, damage to or destruction of housing, interruption or loss of job and income, separation from family, and social connections, or loss of a sense of control or power and feelings of helplessness.

**Table 3:** Selected examples of stress-associated health problems related to disasters with selected supporting literature [modified from Table 1 in Sandifer et al. (11)].

Elevated anxiety, PTSD, PTSS, depression, stress, tension, mental distress	(43–58)
PTSD/PTSS, emotional distress in children	(59–65)
Increased thoughts of suicide among young adults	(53)
Increases in suicidal thoughts, attempts, and suicides	(52, 57, 66–68)
Increased heart problems, elevated blood pressure, stroke, irregular heartbeat, headaches, stomach, and respiratory problems	(47, 48, 69–72)
Increased substance use/abuse	(53, 73–77)
Higher levels of interpersonal/intimate partner/domestic violence	(56, 66, 78–81)
Stress-associated mental (depression and PTSD) and physical health problems related to disaster economic impacts	(82–88)

Economically and otherwise disadvantaged people who may live in places particularly vulnerable to certain kinds of disasters or who may be regularly exposed to polluted air or water [e.g., people in Flint, MI; (89–91)] may feel helpless to improve their situations following disasters and therefore may experience chronic stress resulting in health problems (92–94). Some of these may be referred to as environmental justice situations (95). Similar kinds of effects could be expected among people who depend on natural resources for both a livelihood and way of life (e.g., fishers and fishing communities) when disasters impact their ability to continue to pursue their vocations (96, 97). Those people may feel completely or nearly completely disconnected from processes by which use of the natural resources are controlled in the wake of a disaster, driving feelings of helplessness and hopelessness which may contribute to increased stress, anxiety, and multiple physical health impacts (7, 11). On the other hand, active and meaningful stakeholder participation in disaster recovery may be conducive to increasing feelings of empowerment, decreasing mindsets of helplessness, and improving understanding of what is happening, what is being done and why, all of which can help to counter some negative effects of toxic stress (92–94). Better inclusion of key resource users and consumers from various areas of a community's social and economic life (e.g., fishing, tourism, manufacturing, farming) in pre-disaster planning and post-disaster responses, including research and assessment processes that affect their livelihoods, homes, and families, may help reduce stress and its many negative health impacts.

One important measure of stress associated with negative health effects is Allostatic Load (AL). AL is the cumulative wear and tear on the human body that results from repeated cycles of psychological and physiological stress and recovery, with less and less opportunity for recovery to one's usual physiological state (98–107). Because of its value as a measure of health-impacting stress, AL was included in a recent conceptual model for disaster effects on human health (11). Although chronic and cumulative stress are associated with many, if not most of the long-term health effects of disasters, they are not well measured (or often measured at all) in the types of surveys generally used to gauge health status of individuals and populations following disasters. Methods to measure both pre- and post-disaster stress levels among disaster-affected people will be crucial to understanding the magnitude of the problem, treating it, and to the degree possible reducing or preventing it in future disasters. Our review indicates that much more psychological than physiological data typically are collected following disasters, and a number of standard psychological measures are employed, for example to diagnose PTSD and anxiety, but not always via standardized surveys or clinical assessments. Physiological measures of stress that can be applied in community health assessments are less developed, but the Allostatic Load concept appears to hold a lot of promise. It would be helpful if there was greater standardization in the collection of psychological and physiological data, both pre- and post-disaster.

## Recommendations to Increase Resiliency to Disasters by Reducing Stress Health Effects

To combat and alleviate the various forms of chronic and cumulative stress associated with environmental, technological and other disasters as well as improve community and individual resilience to disasters, we recommend the following actions be considered.

(1) Improve existing disaster behavioral and physical health programs to better address, leverage, and coordinate resources for stress reduction, relief, and treatment in disaster planning and response. Emergency planners and responders recognize that disaster behavioral health (DBH) is an integral part of the overall public health and medical response to any emergency event. The U.S. Department of Health and Human Services (HHS) defines DBH as the psychological, emotional, cognitive, developmental, and social impacts that disasters have on survivors and responders as they respond and recover. Since the majority of DBH activities are accomplished by state, local, tribal, and territorial entities, voluntary organizations active in disaster relief, and individual volunteers, the federal role, largely carried out by the HHS, includes providing preparedness, response, and recovery support to these responders which have direct access to affected communities. HHS resources for DBH are available online^1^ Improvements to DBH and physical health response programs should embrace enhanced recognition of the pernicious, persistent, and omni-present nature of stress-induced disorders. Inclusion and/or greater emphasis could occur at the national level (e.g., via FEMA's National Preparedness Plan and National Response Framework^2^), where childhood trauma related to disasters is already mentioned and supported^3^ and encouraged for consideration at state and local levels. Of the total number that occur globally each year, communities have viewed very few oil spill as disasters„ e.g., the *Exxon Valdez*, DWH, and *Hebei Spirit* oil spills. These spills, and some others, have been studied by a diverse set of social science researchers. Findings have shown that those spills have led to many different types of effects on people, both at the individual and community levels (Figure 3). The regulatory framework for oil spills, as articulated in the U.S. National Oil and Hazardous Substances Pollution Contingency Plan^4^, was revised in accordance with the Oil Pollution Act of 1990 [OPA 90 (108)] to improve preparedness and response to pollution incidents. But this oil spill regulatory framework has a gap. There is a noteworthy absence of any guidance relevant to DBH, stress, or other facets of individual and community well-being, perhaps because of little knowledge of these impacts at the time and the lack of metrics for measuring the extent to which those kinds of impacts could specifically be attributed only to an oil spill.

Following the *Exxon Valdez* and DWH oil spills, researchers studied various elements of DBH and other aspects of health, and after the DWH oil spill major public health research programs were implemented. The Gulf Region Health Outreach Program (GRHOP) was funded by the DWH Medical Settlement and integrated projects focused on primary care, mental and behavioral health, environmental and occupational medicine, and training community health workers to help residents navigate the healthcare system and access needed care. The University of South Alabama's Coastal Resource and Resiliency Center trained community health workers in peer listening to help affected communities, including managing stress (109). But these approaches and resources operate in parallel without explicit connectivity to the existing oil spill preparedness and response framework. There exists no mechanism for oil spill planners and responders to know about, activate, and coordinate the implementation of these kinds of DBH resources, where and when they are available, or how to help make them available during the emergency response phase.

When it is impractical to revise laws or modify national policy, it may be possible for federal authorities to exercise their discretion and adapt the ways existing plans and protocols are implemented within a federal region. Some work along these lines is underway as noted in the Discussion section. Alternatively, it may be more effective to start with emergency plans promulgated and implemented at state, county and municipal levels where most first responders operate and where impacts occur. Initial steps could include engagement with state and local emergency managers, preparedness personnel, public health responders, and natural resource management officials to increase their understanding of the important roles that stress plays in both short- and long-term health impacts of disasters. Engagement efforts should focus on identifying the likely to be affected communities and individuals. This would be consistent with effective risk communication practice, defined by the National Academy of Sciences (110) as an interactive process of exchange of information and opinions among individuals, groups, and institutions concerning a risk or potential risk to human health or the environment. Risk communication is a fundamental component of risk management; it involves risk managers listening to the risk perceptions, questions and concerns of a broad range of potentially impacted individuals and communities. Expertise and information can have a large impact on risk perceptions which, in turn, can help reduce stress and strengthen individual and community resilience.

An important consideration in this regard is preparedness and post-disaster services and funding. When stress (and AL), which some regulatory and legal systems may categorize as personal injury, is omitted from the conditions eligible for grants, claims, and damage compensation, then it is essentially impossible for authorities to allocate funds to support stress recovery. Thus, it is vitally important to find or create an appropriate regulatory space to include stress so it can become an integral part of pre- and post-disaster risk communication and recovery processes. Inclusion should provide specifically for increased capacity to provide services for diagnosis, treatment, monitoring, and recovery of stress-related/caused disorders, e.g., AL as an explicit component of DBH protocols and practices, not just in the immediate aftermath of disasters but as part of disaster preparedness. Protocols should address the post-disaster duration of services, recognizing the variability in individual needs and speeds of recovery. For example, behavioral health recovery, and associated physical health impacts, may take longer than recovery from immediate physical injury, e.g., broken arm. Along with development of diagnostic tools and the collection of essential data as elaborated in recommendation 2, adding stress-related disorders and long-term duration for DBH services to protocols would be a critical step in increasing resiliency among individuals and communities who are likely to experience severe disaster-induced AL.

(2) Emphasize pre- and post-disaster collection of relevant biomarker and other health-related data to provide a baseline of health status against which disaster impacts could be assessed, and continued monitoring of these indicators to evaluate recovery. Numerous researchers have identified the use of biomarkers to assess health effects of disasters and other traumatic events and the collection of these and other indices of mental and physical well-being both pre- and post-disaster as an important area of research, for developing baseline data against which to evaluate disaster impacts and for health recovery assessments following disasters [e.g., (11, 111–114)]. The development of biomarkers and other measures of stress is principally the province of entities that fund and do research. However, to become operationally meaningful, the costs for implementation of collection, both before disaster to provide baseline data, and after disaster use of biomarkers in assessing health effects should be part of disaster planning, management, recovery funding.

As noted previously, AL can provide a measure of stress related to negative health outcomes. It is typically assessed by a combination of endocrine, immunological, metabolic, and other biomarkers, such as cortisol, cholesterol, blood pressure, pulse rate, and body mass index among others, that represent a variety of biological functions (99, 106, 107). However, it is not yet standardized for routine use in health assessments, nor are AL data regularly collected from individuals or groups in advance of anticipated disasters. Because of the highly complex nature of the interacting biological components that make up AL, its measurement is still under development (115). Nonetheless, Buckwalter et al. (115) demonstrated important progress, using a combination of 20 biomarkers in a computational model. Rusiecki et al. (114) noted the potential for using the Department of Defense Serum Depository of more than 50 million serum samples collected since 1985 from members of the military and continuing forward for evaluating biomarkers for exposure and biological effects related to oil spill exposure. Having a baseline of this type will be significant to address effects of future disasters where the US Coast Guard or other military units may be called in to assist. Unfortunately, we are not aware of similar extensive biomarker databases for civilian populations. Attention is needed to begin development of such databases in areas that are particularly risk-prone, such as the Gulf of Mexico states, and to development of operational AL metrics and other measures of disaster-induced stress so these can be incorporated into disaster preparedness and response plans. Support for biomarker and database development is urgently needed.

(3) Enhance capacity of science and public health emergency responders. First responders to disasters are usually police, fire fighters, military personnel, emergency managers, and emergency medical service (EMS) personnel. These specially-trained responders work on the front lines of a disaster to mitigate immediate risks to human health and property, often at considerable risk to themselves. They are essential components of modern disaster response capacities, and these dedicated, trained, and certified first responders are crucial elements of community resilience (116). In addition to this immediate trauma support, there is a widely recognized need for better scientific and public health responses to disasters, to improve ability to detect and mitigate health threats such as pollution and disease, begin dealing with the psychological and other health threats that are of major concern after the urgent, life-threatening issues are addressed, and be a source of trusted information for the public (15, 117, 118). Psychological first aid is also required (119, 120) to help mitigate the initial mental distress caused by a disaster. To better serve disaster-affected communities, the US needs to develop integrated, rapid disaster research response capabilities that are (a) coordinated across agencies, (b) liaise with emergency workers, volunteers, communities, and disaster managers at various geographic levels, (c) target areas of concern to affected people, and (d) communicate trusted, evidence-based information to the public (11, 23, 118, 121, 122). Some important progress has been made in this regard with the National Institutes of Environmental Health Sciences (NIEHS) new Disaster Response Research (DR2) Program (118)^5^ Referring to emergency-driven research, Miller et al. (118) noted that “Our inability to perform timely research to inform the community about health and safety risks or address specific concerns further heightens anxiety and distrust,” and thus further compounds stress from a particular event. That problem, along with recognition of shortcomings in the public and community health aspects of the response and in communication of research targeting health-related questions and concerns specifically related to the DWH oil spill, apparently was part of the motivation for development of the DR2 program. The DR2 effort focuses on six primary objectives: research questions and priorities, access to data collection tools, improved capability for rapid data collection, trained disaster researchers, integration into disaster management systems, and inclusion of public health, academics, affected workers, and the public.

While the DR2 is a great step forward, much remains to be done to integrate more knowledge into response actions across agencies and with academic institutions, public health responders, emergency managers, and others to achieve the kind of real-time, expert-informed disaster research response envisioned by McNutt (122). Some areas of particular concern include identifying a public health emergency workforce, maintaining it in action-ready state over the long term and between emergencies, and building and sustaining robust partnerships with a broad range of academic, government, business, and community participants in regions that are known to be particularly susceptible to hurricanes, wildfires, or other kinds of disasters. Inclusion of a prominent community participatory element is crucial for both effective public health response and research following disasters (123).

(4) Use natural infrastructure to minimize disaster damage. The use of more natural infrastructure instead of, or in combination with built or “grey” infrastructure to provide coastal protection from hurricanes, other extreme weather events, and sea level rise is rapidly becoming accepted by coastal managers and others (124, 125). This green + gray strategy is based on extensive scientific evidence that demonstrates the significant roles that intact natural coastal features such as wetlands, dunes, and islands, can play in storm wave attenuation, erosion reduction or prevention, shoreline stabilization, flood defense, and other protective characteristics (126–137), conservation of biodiversity (138), and perhaps also in protecting in human health (139). The economic values of these nature-based defensive mechanisms can be substantial. For example, Narayan et al. (140) estimated that the presence of coastal wetlands resulted in avoidance of approximately $625 million in flood damages that could have occurred with Hurricane Sandy. A substantial ongoing effort to help coastal communities take advantage of the economic and environmental benefits of natural infrastructure is being undertaken by the Coastal Resilience partnership ^6^ led by The Nature Conservancy in collaboration with federal agencies, academic institutions, non-governmental organizations, and international partners. To strengthen protection of coastal communities and property, coastal permitting guidance [e.g., (141)] and recovery plans could articulate that use of natural infrastructure where possible is preferred, whether used alone or in combination with engineered options. This nature-based guidance also should be preferred in pre-and post-disaster recovery projects, e.g., those funded by FEMA. Undertaken by the US Army Corps of Engineers, or supported by RPs in pollution incidents.

(5) Expand the geography of disaster response and relief to better incorporate the displacement of affected people. The scope of recent natural and Natech disasters in the US and globally points to a need to consider disaster impacts at much larger geographic and longer time scales than has typically been the case. For example, Hurricane Katrina affected a land area of Louisiana and Mississippi larger than that of the United Kingdom and resulted in the evacuation of some 485,000 residents, including essentially the entire population of the city of New Orleans (79, 142); the Fukushima tsunami and nuclear plant meltdown resulted in forced evacuation of over 97% of the ~76,000 people living within a 20-km radius of the plant (67); Hurricane Harvey directly affected some 13 million people with 32,000 being displaced (143); Hurricane Maria devastated the entire island of Puerto Rico and impacted all of its population in some way. Although no evacuation was possible for Puerto Rico due to its island nature, Melendez and Hinojosa (144) estimated that by 2019; Puerto Rico may lose over 470,000 people or about 14% of its population to a Hurricane Maria-driven exodus.

Ethnic minorities, older people, females, and those with chronic health problems and/or significant economic challenges are typically the most affected by health impacts, stress, and intimate partner violence during and following disaster evacuations and displacements (44, 66, 67, 78, 79, 142, 145–150). In the case of Hurricane Katrina and the Fukushima accident, the lack of medical care and support during and immediately following evacuations resulted in substantial mortality, especially among vulnerable people. In addition to ensuring that medical care is available during evacuations, a concentrated effort to rapidly return displaced persons to stable housing and economic situations (jobs) likely would have a strongly positive impact on health (35, 151). While extended displacements of disaster victims typically have negative health impacts, there are occasions when insights gained from previous diasporas may prove helpful. For example, many Puerto Ricans who no longer resided on the island provided critical support for those affected by Hurricane Maria (152). Opportunities to make better use of such dispersed social resources should be included in disaster planning, along with long-term commitments to provide health care options and suitable housing and income-generating opportunities for displaced individuals until they can return to their homes and work.

(6) Utilize nature-based treatment and nature-exposure to the greatest extent practical to alleviate pre- and post-disaster stress effects such as anxiety, depression, and loss of cultural and place identity. It is widely recognized that people derive a broad range of psychological and physiological health benefits from experiencing more natural, “green,” and biodiverse areas, including those near water bodies and coasts (11, 139, 153–159). These health-enhancing effects are key ecosystem services provided by nature (160). However, environmental disasters may reduce or degrade these ecosystem services with concomitant increases in acute, chronic, and cumulative stress in humans and associated negative health outcomes (11, 139, 161).

Although protection of life and property are first-order concerns, alleviation of disaster-caused stress should also become an early and ongoing consideration in disaster preparedness and response. In a comprehensive review of ecotherapy, Summers and Vivian (160) present extensive, compelling evidence for positive effects of nature exposure in numerous areas of health care, including medical recovery (e.g., blood pressure heart rate, recovery from surgery, cardiopulmonary rehabilitation); restoration; reduction of pain, stress, and post-traumatic stress; improvement of mood and symptoms of ADHD (attention deficit hyperactivity disorder) and dementia; reduced obesity; improved vitamin D levels; and enhanced cognition, creativity, and development in children. They concluded that “Clear and abundant evidence demonstrates that interaction with nature affects not only well-being but health throughout life. This evidence suggests that people, who as children strongly interact with ecosystems and environment live longer with better quality of life.” In addition, exposure to and opportunities for quiet contemplation in natural settings and coastal areas are known to promote mental relaxation and reduce stress and anxiety (153, 162–164).

Building on the ecotherapy concept, some modern physicians and public health practitioners are promoting expanded use of nature-assisted therapies to help improve health, alleviate mental distress, and treat post-traumatic stress disorders (165–170). The US National Park Service has established a Park Rx program^7^, as have Cornell University^8^ and the College of William and Mary and the surrounding community of Williamsburg, VA^9^ The University of Maryland is also in process of developing such a nature-based health program, with a planned start in 2019 (Ariana Sutton-Grier, personal communication, cited with permission).

While ecotherapy is not yet widely practiced and prescribed (171, 172), it is growing in acceptance and appreciation. Based on some successes of ecotherapy in treating or at least alleviating a variety of health problems, including trauma-related stress, it would seem logical to include it as much as possible in disaster response and recovery activities. For coastal communities whose residents rely upon nature-based relaxation pre-disaster, it may be especially important for their resilience to provide ecotherapy opportunities for them post-disaster such as green spaces, parks, gardens, water bodies and coasts, and wetlands. Opportunities where affected disaster victims could receive ecotherapy would be beneficial and should be incorporated into disaster recovery plans at all levels.

(7) Review disaster laws, policies, and regulations to identify opportunities to strengthen public health preparedness and responses, specifically including stress-related impacts, better engage affected communities, and enhance provision of health services. With the exception of oil spills, most disaster planning and relief efforts from the US federal government are carried out under the authorities established by the Stafford Act (173); P.L. 93-288 as amended. This law provides for comprehensive disaster preparedness, response, and recovery plans at federal, state and local levels, financial and other assistance to affected jurisdictions, emergency housing relief, and much other critical support leading up to and following emergencies and major natural catastrophes or fires, floods or explosions, regardless of cause, that receive a formal request for federal assistance from a state governor and a declaration by President of the United States. The Act also states in Section 311 that appropriate property loss insurance should be obtained and maintained. Within the U.S. Department of Homeland Security (DHS), FEMA administers assistance programs that are authorized under the Stafford Act, and 32 Core Capabilities are identified to achieve the National Preparedness Goal. Two core capabilities—Public Health, Healthcare & Emergency Medical Services and Health and Social Services- include mental and behavioral health. Following the terrorist incidents of recent years, DHS and FEMA recognized the need to plan for Complex Coordinated Terrorist Attacks (CCTAs) and their disastrous impacts on communities. To this end, FEMA has a grant program and guide to help communities prepare for CCTAs^10^ 7, (174). Behavioral and mental health services for survivors are noted. These programs and core capabilities are vital components of the overall US disaster preparedness capacity. The fact that behavioral and mental health are noted goes a long way toward building resilience, both within government at all levels and in communities across the country.

For catastrophic oil spills, OPA 90 (as amended), enacted following the 1989 *Exxon Valdez* spill in Alaska, considerably strengthened preparedness and response capabilities at all levels. Although very few oil spills become true disasters, when this becomes the case, as with the DWH oil spill, OPA 90 provides the framework for a substantial response, including environmental damage assessments and levying of damage claims against the responsible party(ies). While both the Stafford Act and OPA 90 establish robust structures for disaster response, noteworthy gaps or disconnects related to stress-associated impacts have become apparent in the two decades since their enactment.

For most disasters governed by the Stafford Act, shortcomings include highly bureaucratic processes that are often difficult and time-consuming to work through, resulting in excruciatingly slow delivery of certain kinds of assistance (e.g., housing), a need for revitalization of the Public Health Emergency Fund including better coordination among Federal agencies involved and more funding (175), much more attention to long-term recovery (176), more attention to building state and local capacity to cope with disasters including housing (177, 178), and ongoing delivery of health care and health support at needed geographic, temporal, and social scales. While the Stafford Act does not require the federal government to provide housing or medical assistance, it usually does, but often not in timely, comprehensive, or consistent fashion or for the extended periods of time necessary for full recovery as the experiences of Hurricane Katrina so horrendously demonstrated. In response to direction from President Obama, FEMA (34) established a National Disaster Recovery Framework^11^ (34) (that was released just before Hurricane Sandy hit the US. The framework encompasses six important recovery support functions, including housing and health and social services. Unfortunately, the delivery of housing, food and water, electrical power, and medical and health services is still inadequate relative to the needs as again painfully revealed following Hurricanes Sandy, Harvey and Maria. Solving these problems, or at least being better prepared for the next disaster, will require stronger leadership; more funding; improved coordination at all levels of government and response; elimination, reduction, and streamlining of bureaucratic requirements; and a new commitment on the part of states, local governments, and individuals to do their parts to develop and implement disaster housing and medical support plans rather than depending primarily on the federal government. A particularly important deficiency is the apparent failure to include disaster-induced stress as a root cause of many of the significant, long-term mental, and physical health problems associated with disasters. Addressing stress should be a prominent feature of federal, state, and local disaster preparation and recovery plans, considering the long periods of time typically required to deal with trauma-induced health problems.

For oil spills, the requirements of the OPA 90 could be adapted to strengthen connections with communities affected by oil spills when they result in disastrous human dimension effects (42). Current oil spill contingency planning in the US fails to recognize or operate through local networks that could more effectively enhance inherent (i.e., adaptive) resilience (38), or to coordinate well with activities undertaken under the Stafford Act provisions (179). It does little to reveal potential impacts on local communities and individuals that may not be related to direct oil exposure, such as stress. The provision of external resources and knowledge is necessary for communities to adapt and be resilient to environmental changes caused by oil spills (180). In working with spill authorities and specialists during preparedness activities, communities can learn more about spill impacts, what they can expect from the federal government, and what they can and should do to help themselves and the responders in the event of a disaster, and thereby strengthen their adaptive capacity for resilience. Section 4202, National Planning and Response System, of OPA 90, established Area Committees and requirements to develop Area Contingency Plans and strengthen oil spill preparedness at the Area level, which typically includes multiple counties in one or more states. Because the catalyst for oil spill laws and regulations was to protect the environment from pollution, human dimension impacts were addressed in the limited context of public and worker safety and compensation for socio-economic damage directly caused by pollution. There is no means to address those related to personal injury, e.g., stress and behavioral health, or long-term injury not caused directly by toxicity or physical injury, other than law suits. Yet, studies have shown that post-spill litigation exacerbates individual and community impacts (96, 97, 181–183) and that those affected in a spill area have incident-specific questions and concerns that, when left unanswered, contribute to behavioral health impacts (184).

Following the DWH oil spill, US Coast Guard headquarters issued a new Area Contingency Planning Process Job Aid (185) to address preparedness gaps at the area level, i.e., generally within a state. This guidance encourages pre-spill planning to be more collaborative with the full range of stakeholders, including other users of natural resources such as fishers and the tourism industry. Trusted working relationships developed pre-spill through mutual respect may reveal novel ways to more effectively address new questions, concerns, and risk perceptions which invariably emerge during response (186). Currently, in Virginia a new annex to the Area Contingency Plan is under development which will directly seek input from communities to identify key initial actions, local resources, and specific questions and concerns to incorporate into a geographic-specific Eastern Shore Annex. In short, this is an adaptation to engage geographically-isolated, eco-dependent communities in a meaningful way pre-spill to transfer knowledge, build trusted relationships, and develop an adaptive capacity for resilience should an oil spill occur in their area. Similar efforts could be taken in every region of the country under OPA 90 and through other avenues (e.g., the National Response Plan) for disasters of all other types and could take into account stress, DBH, and other types of personal injury, which are omitted from coverage under the Oil Spill Liability Trust Fund. To advance this capability, at the time of this writing, the National Academy of Sciences' Gulf Research Program and Sea Grant Oil Spill Science Outreach Program are planning five regional workshops to focus on the public health, social disruption, and economic impacts of oil spills^12^.

(8) With community participation, develop and institute equitable processes pre-disaster for dealing with damage assessments, litigation, payments, and housing. One of the most damaging long-term impacts of disasters that result in significant damage to livelihoods, housing, and customary ways of life and that produce untold stress is the erosion of confidence among the affected public in the responsiveness, fairness, and transparency of processes, agencies, institutions, companies, and even individual leaders involved in adjudication and payment of damage claims under OPA 90, the provision of financial and housing support under the Stafford Act, and the processing and payment of insurance claims. Problems of this nature related to oil spills have been described and researched in detail following the 1989 *Exxon Valdez* oil spill in Alaska and the 2010 DWH oil spill in the Gulf of Mexico. These and previous incidents demonstrate how the ensuing litigation, disagreements, long-term nature and, at least to some, the questionable fairness of claims processes pitted victim against victim as well as against RPs and government agencies resulting in long-lasting stress and associated health problems. As a result, new concepts of enduring psychosocial impacts of certain kinds of disasters, especially technological ones, have been proposed, such as “corrosive communities,” i.e., communities characterized by “social disruption, lack of consensus about environmental damage, and general uncertainty” (187), “recreancy,” referring to the failure of individuals and/or organizations to do what they were supposed to do (188), and “renewable resource communities,” those that are heavily dependent on natural resources impacted by a disaster (7). Ritchie's use of social capital theory to integrate them helped pave the way for a more detailed evaluation of the lingering psychosocial impacts of technological disasters (9, 187, 189).

Problems with the DWH oil spill claims process elaborated by Mayer et al. (190) and Ritchie et al. (191) demonstrate that these problems have not been resolved, despite considerable information from years of study on the *Exxon Valdez* accident. Similarly, the impacts of the huge natural disasters of Hurricanes Katrina, Harvey, and Maria were exponentially increased by epic institutional failures as was the technological disaster involving drinking water in Flint, MI (90, 91). One only has to review the extensive literature on Katrina and the fact that New Orleans is not yet back to “normal” nearly 15 years out from the storm, as well as the continuing suffering in Houston and especially in Puerto Rico to conclude that these are American humanitarian crises of the first order. Michaud and Kates (57) noted that the Puerto Rico power outage was “the largest blackout in American history” and the power grid, even where repaired, remains substandard. Houston, where much of the damage to homes was likely uninsured (143, 192), has only just developed a housing recovery plan in response to the approximately 29% of its population that was likely affected by Hurricane Harvey flood damage to homes^13^ This plan is scheduled to be launched in fall 2019, a full year after the flooding^14^ Similar efforts are apparently underway for Puerto Rico, but based on previous experience elsewhere, the sad state of housing there even before Hurricane Maria (193), and the widespread lack of insurance (194), getting funding for housing repair or replacement and to support economic recovery in the hands of Puerto Ricans is likely to take a long time. Such delays in getting housing recovery funds to those impacted cause considerable stress among affected people, at least some of which could have been avoided if adequate housing relief and recovery plans were prepared in advance and with local input. At the same time, one must recognize the very real potential for fraud in damage claims, cleanup expenses, housing, and other compensation based on experiences with Hurricane Katrina, previous oil spills, and other disasters^15^ If repeated in Texas and Puerto Rico, such incidents may increase recreancy both among those directly affected by the disasters and citizens whose taxes fund government relief programs. Similarly, failure by individuals to purchase and maintain appropriate insurance, as well as difficulties experienced by survivors of previous storms even when insured, in receiving what they perceived to be fair compensation through inefficient and delayed processing or rejection of claims, likely have exacerbated the stress levels experienced by survivors. The public needs assurance that procedures to reduce (preferably eliminate), identify, and prosecute fraud are included in plans and operating procedures from the outset and clarity about these processes. Similarly, poorly crafted and managed, and in some cases misleading (e.g., Flint, MI), disaster communications can lead to loss of public faith in governmental institutions and leaders, and contribute to poorly informed opinions, which lead to subsequent inaction or actions inappropriate to mitigate the actual risks. Thus, risk communications need to be clear, consistent, and credible, to the degree possible crafted before an incident so they are ready to distribute with situation-specific refinement, written appropriately for different audiences taking account of gender, ethnicity, age, and socio-economic differences, and delivered by trusted sources (195).

We recommend that communities in areas known to be vulnerable to natural or technological disasters, e.g., with a history of frequent threats from hurricanes or proximity to chemical manufacturing or energy facilities, consider developing, implementing, and regularly updating processes for requesting federal assistance and potential damage claims and compensation payments well in advance of disasters, including ways to prevent and detect fraud. While many communities may be involved in risk management and have local emergency planning committees^16^, there is room for improvement, especially with regard to preparedness funding and post-incident financial assistance. We further recommend these processes include: easy access to reliable and understandable information; explicit and continuing recommendations for insurance purchase; clear, standardized guidance as to eligibility and claims submission, whether for damage claims related to an oil spill or government assistance; an independent or third-party entity, preferably locally-based, to serve as a community liaison and help coordinate and/or process damage claims where a responsible party is involved; and opportunity for public input into the original design and adoption of a process and later for its implementation. For other kinds of disasters, e.g., intentional acts of terror or arson, various resources are available but may be unknown until needed, e.g., grief counseling and National Compassion Fund created by the National Center for Victims of Crime in the U.S.

## Discussion and Conclusions

Disasters are a recurring fact of life, and major incidents can have both immediate and long- lasting negative effects on the health and well-being of people, communities, and economies. Reducing the likelihood and severity of disaster impacts through increased resilience at both individual and community levels are widely sought-after goals of disaster preparedness, response, and recovery plans. As Hurricanes Katrina, Rita, Sandy, Harvey, Irma, and Maria, the catastrophic DWH oil spill, and other recent disasters around the world have demonstrated, humans usually respond rapidly and with a massive outpouring of effort and material in the immediate aftermath of such disasters. But, attention and support rapidly wane (123), leaving long-term issues of high stress levels, impaired mental and physical health, disrupted communities and social structures, damaged or destroyed homes and means of employment, and senses of helplessness, anxiety, and anger. What the U.S. needs are more robust immediate- and intermediate-term responses coupled with sustained efforts that do not weaken over time but carry on addressing health and well-being issues over years to decadal time frames. Unfortunately, as recent disaster responses show, the US is not yet able to deal with the full range of public health, infrastructural, and other problems associated with disasters as effectively as desired, even though emergency and disaster planning and response processes are well developed and reasonably adaptable.

The government response to Hurricane Katrina was characterized as “perhaps the biggest failure of public administration in the nation's history” (196). The reaction was so poor that, reacting to the “horrifying images on television” and the perceived massive malfunctioning of government at all levels, for the first time ever numerous international non-governmental organizations provided humanitarian aid inside the U.S. (197). Following Katrina, substantial efforts were made to strengthen FEMA and the overall US disaster response capacity. As a result, governmental response to Hurricane Sandy in 2011 was noticeably improved [e.g., see (198)^17^, (199)], but many health-related problems persisted. Some members of the public, especially the elderly trapped in high-rise buildings without power and medicines, were again left feeling helpless for considerable periods of time (199). Not surprisingly, some of the same themes of failure of coordination, leadership, etc. noted following Katrina were reflected in FEMA's Sandy after-action report (200). More improvements were noted in government's response to Hurricanes Harvey and Maria, based in part on lessons learned from Katrina and Sandy. Disaster response plans had been improved, staff were better prepared with pre-positioned resources, and the agency was much more willing to accept help from non-federal partners including non-governmental organizations (e.g., the Red Cross), private business and others as well as state and local government entities (12). A commitment to more inclusiveness was reflected in the agency's after-action report (17) where the agency referred to the significant contributions that “brave residents,” NGOs, the private sector, and others made, along with state and local emergency responders and federal workers to the overall disaster response in both Texas and Puerto Rico. The report also included continued strengthening of collaborations and state response capacities as key steps for the future. Again, however, there were high order failures, especially in dealing with the catastrophic effects of Maria on Puerto Rico^18^ As a result, FEMA recognized that it must be ready to cope with simultaneous high-magnitude disasters and specifically recommended that it “should work with partners and the White House to revise the current National Response Framework and, as required, the Response Federal Interagency Operational Plan to emphasize stabilization of critical lifelines…such as power, communications, health and medical [emphasis added], food and water, wastewater, and transportation,” and housing (17). The report further states “The rapid stabilization of the lifelines would be the organizing principle for the doctrine.” We hope that FEMA and its federal interagency partners and the states will follow up on this recommendation with rapid, positive action. However, it is disappointing that this is one of the few mentions of “health” in the after-action report and “stress” is not mentioned at al. If, as FEMA proposes, the U.S. should build a “culture of preparedness,” we recommend that a revised Framework and Interagency Response Plan incorporate protection and recovery of human health and well-being, including of disaster workers, as its primary goal, with stabilization of the listed lifelines as means to that end. This recommendation follows findings from recent research in the Gulf of Mexico region that highlight the potential that provision of integrated mental and physical health care in disaster-prone areas has to strengthen resilience among individuals exposed to severe disaster-related stress (201).

As Kettl (196) emphasized, every disaster is different, and we cannot succeed in managing new disasters solely by preparing for what we have seen in the past. This is particularly true in this era of accelerated climate change. Emergency response and disaster systems learn by experience and modify plans and procedures with what is learned. Nonetheless, what we saw with the nation's response to the 9–11 terrorism event, more cogently in the run-up to and aftermath of Hurricane Katrina, and have continued to see with Hurricanes Harvey, Irma, and Maria is what the 9–11 Commission [quoted in Kettl (196)] described as a “failure of imagination.” Apparently, we as a society had not imagined that such events, devastation, and humanitarian crises could occur here, and thus did not adequately prepare for them. In the US, our laws and regulations are often created or modified reactively rather than proactively. At least part of an ongoing challenge is how to proactively identify and address wicked problems, i.e., those that are complex, unpredictable, open ended, or intractable and for which solutions may create other problems (178, 196, 202–204). The U.S. continues to be surprised by many disasters, and therefore lacks the perfect combination of resources needed immediately following each disaster to mitigate threats and impacts to affected communities. Political will and public attention can be too fleeting to sustain resourcefulness and help systems be ready for whatever comes no matter whether we have imagined and planned for it or not. That is not to say that all that has been done to create and implement disaster response systems is weak; it is not, in fact it is comprehensive, but still insufficient to deal with the complex, wicked disasters which occur. We need to take what we have and *imagine* a disaster response system that is in place and ready to go always; that is cooperative and collaborative with residents and communities in the interregnum between disasters, not just the immediate before and tragic after periods. By inviting communities to participate in developing drill and exercise scenarios, we can learn about risk-laden disaster situations which worry them but may be outside the thinking of planners and responders (205). Through proactive collaboration, the process can become transparent, participatory, and trust-building so that people have confidence that, as a disaster looms over them, all levels of government and other organizations are working together to ensure their health and safety. To benefit affected communities and foster resilience, the system should continue to be supportive and responsive throughout the long tail of recovery. Through effective risk communication well ahead of time of need, communities should be educated about existing government, community, private, and other resources as well as self-help preparation and responsibilities so individuals and communities are equipped and fortified to react effectively on their own to unforeseen circumstances. What we are talking about is a preparedness and response system, with plans, organization, communities, and individuals, that would have a formally recognized component of adaptive management to respond to *and promote* resilience to impacts of disasters which were not previously imagined. Adaptive management is a decision-making process that works to reduce uncertainty by continuous monitoring and making adjustments to actions over time (206). This would be a large stretch of the system, but it is not out of reach.

Understanding the relationships of the stress-related issues discussed in this paper is still being revealed by research and experience. Only in recent years have research funders begun to emphasize trans-disciplinary research and syntheses of findings across the silos of study in the physical, social, and health sciences. Mental and behavioral health impacts are notably complex, often viewed as subjective, and still vulnerable to social stigma. Teasing out the contributions of stress, e.g., AL, to physical and behavioral health is also evolving. Applying in real time, trans-disciplinary research findings in a coordinated, interagency process is complicated. Baseline data of all kinds are lacking to provide defensible metrics and acceptable evidence of post-disaster impacts, including biometrics. Therefore, a continuing need exists to focus on developing protocols and approaches to mitigate stress-related impacts from disasters, which can be incorporated into preparedness and response plans.

So, how do we imagine that a spotlight on reducing negative stress-association health outcomes could improve individual and community resilience to disasters? Patel et al. (25) suggested that focusing on individual components of resiliency may be more effective for implementation of resilience efforts that are adaptive than trying to do all things at once. In this light, Buckner et al. (207), in describing the Gulf Region Health Outreach Program established following the DWH incident, noted that in part the program focused on strengthening community resilience by improving access to, knowledge of, and infrastructure for health care. Here we posit that identifying health and well-being as the primary, central focus for disaster management—along with identification of stress as a root cause of both short- and long-term impacts to the health of individuals, groups, and communities—could be a crucial first step toward increasing disaster resilience at both community and individual levels. This step, followed by implementation of the eight actions we describe herein—(1) improve existing disaster behavioral and physical health programs to better address, leverage, and coordinate resources for stress reduction, relief, and treatment in disaster planning and response; (2) emphasize pre- and post-disaster collection of relevant biomarker and other health-related data to provide a baseline of health status against which disaster impacts could be assessed, and continued monitoring of these indicators to evaluate recovery; (3) enhance capacity of science and public health early-responders; (4) use natural infrastructure to minimize disaster damage; (5) expand the geography of disaster response and relief to better incorporate the displacement of affected people; (6) utilize nature-based treatment to alleviate pre- and post-disaster stress effects on health; (7) review disaster laws, policies, and regulations to identify opportunities to strengthen public health preparedness and responses including for stress-related impacts, better engage affected communities, and enhance provision of health services; and (8) with community participation, develop and institute equitable processes pre-disaster for dealing with damage assessments, litigation, payments, and housing—would go a long way toward working collaboratively to enhance resilience and improve the ability of planners to develop resilience efforts that can be implemented now and be more adaptable to future conditions and events.

Hamlin et al. (208) recently described an “adaptive gradient framework” for coastal resilience. Targeting the actions recommended here through a process like their cross-disciplinary, facilitated workshop approach and involving public, mental and physical health practitioners, ecologists, lawyers, economists, and representatives of the affected publics, along with disaster preparedness and response specialists, could lead to significant improvements in community resilience and lessen health disaster health impacts. Inclusion of greater emphases on empowering and building capacity of local community organizations could help to minimize social disruption and more rapidly repair social connectivity. In the end, people need to be confident that they themselves, government, and other responders are as ready as possible, with plans that are familiar, transparent, easy to understand and act, and that the entities in whom they place their trust will be with them for the long haul.

## Author Contributions

PS provided the initial concept and did the majority of literature review and writing. AW provided key ideas and contributed significantly to the review and writing of the paper. Both authors support the findings and recommendations of the paper.

### Conflict of Interest Statement

The authors declare that the research was conducted in the absence of any commercial or financial relationships that could be construed as a potential conflict of interest. The reviewer JS declared a past co-authorship with one of the authors PS to the handling editor.

## Footnotes

^1^https://www.phe.gov/Preparedness/planning/abc/Pages/DBH-federal.aspx.

^2^https://www.fema.gov/media-library-data/1466014682982-9bcf8245ba4c60c120aa915abe74e15d/National_Response_Framework3rd.pdf

^3^https://www.nctsn.org/what-is-child-trauma/trauma-types/disasters

^4^https://www.epa.gov/emergency-response/national-oil-and-hazardous-substances-pollution-contingency-plan-ncp-overview

^5^https://dr2.nlm.nih.gov

^6^http://coastalresilience.org

^7^www.Parkrx.org

^8^https://naturerx.cornell.edu/default

^9^https://www.wm.edu/news/stories/2017/park-rx-day-celebrating-natures-profound-power.php; http://www.gwaparkrx.com/park-ambassadors.html.

^10^https://www.fema.gov/media-library-data/1532550673102-c4846f270150682decbda99b37524ca6/Planning_Considerations-Complex_Coordinated_Terrorist_Attacks.pdf

^11^https://www.fema.gov/pdf/recoveryframework/ndrf.pdf

^12^https://gulfseagrant.org/oilspilloutreach/collaborative-workshop-series/

^13^https://dfw.cbslocal.com/2018/06/08/houston-draft-plan-harvey-housing-recovery/.

^14^http://www.houstontx.gov/housing.

^15^http://www.nydailynews.com/news/national/justice-department-warns-disaster-fraud-irma-harvey-article-1.3495981#; https://www.pbs.org/newshour/nation/authorities-alert-fraud-hurricane-harvey.

^16^LEPCs https://www.epa.gov/epcra/local-emergency-planning-committees

^17^https://www.nytimes.com/2012/11/05/opinion/krugman-sandy-versus-katrina.html

^18^https://www.economist.com/briefing/2018/04/14/america-has-let-down-its-puerto-rican-citizens; https://www.theatlantic.com/science/archive/2017/10/what-happened-in-puerto-rico-a-timeline-of-hurricane-maria/541956/
